# Seasonal changes in the force velocity sprint profile of Spanish youth football players across age categories

**DOI:** 10.1038/s41598-025-89501-3

**Published:** 2025-04-10

**Authors:** Antonio Hernandez-Martin, Jorge Garcia-Unanue, Fabio R. Serpiello, Antonio Alonso-Callejo, Jose Luis Felipe, Leonor Gallardo, Javier Sanchez-Sanchez

**Affiliations:** 1 Deparment of Sports Sciences. Faculty of Medicine, Health and Sports, Universidad Europea de Madrid, Villaviciosa de Odón, 28670 Spain; 2 IGOID Research Group, Physical Activity and Sport Sciences Department, University of Castilla-La Mancha, Toledo, 45071 Spain; 3 School of Health Medical and Applied Sciences, Human Exercise and Training Lab, Central Queensland University, Rockhampton, Australia

**Keywords:** Football, Force, Velocity, GNSS/GPS, Young, Developmental biology, Health care

## Abstract

The purpose of this study was to investigate the changes in the force-velocity (F-V) profile and 30-metres sprint times of youth football players over a competitive season and across different age groups. Sixty-four players were categorized into five age groups (Under-10 (U10), Under-12 (U12), Under-14 (U14), Under-16 (U16), Under-18 (U18)) and assessed at three time points during the season: the pre-season (P1), mid-season (P2), and end season (P3) using GNSS/GPS technology. Results showed that the theoretical maximum force (F0) increased by the end of the season compared to the baseline in U14, U16, and U18 (*p* < 0.05; ES: 1.92–5.19) and was also higher at the end compared to mid-season in U14 players (*p* < 0.05; ES: 3.01). The theoretical maximum velocity (V0) was significantly higher at the end of the season compared to the baseline in U12 (*p* < 0.05; ES: 2.61) and mid-season in U12 and U16 players (*p* < 0.05; ES: 1.70–2.37). U10 and U12 showed lower F0 and V0 values compared to older players across all periods. The study concludes that both the timing of the season and the age influence the F-V profile, with older age groups showing greater improvements. These findings provide valuable insights for optimizing training programs tailored to the seasonal changes and age of football.

## Introduction

Football is an intermittent sport characterized by a combination of high-velocity actions and low-intensity breaks^1^. High-intensity actions, such as accelerations, decelerations, and sprints over short and long distances, have significantly increased in modern football, up to approximately 35% more than in previous seasons^2^. These actions are becoming the most frequent events preceding crucial moments in a match, such as scoring a goal^3^. Consequently, improving sprint performance has become a major training objective^4^. Sprinting involves a player’s forward acceleration, which generates high horizontal force against the ground when initiating the sprint^5^. This relationship between maximum force production capacity and horizontal velocity has been described by various authors as the force-velocity (F-V) profile^6,7^. This profile is based on the force-velocity and velocity-power relationships of the neuromuscular system in the lower limbs. Additionally, because the F-V profile only considers horizontal force when sprinting at maximum velocity, the force an athlete can apply is lower the more velocity it reaches^8^.

The variables obtained from the F-V profile include theoretical maximum velocity (V0), theoretical maximum force (F0), the slope of the F-V relationship (F-V slope), maximum power (Pmax), the decrease in the ratio of horizontal to resultant force (DRF), and the maximum horizontal-to-resultant force ratio (RF peak)^7^. It is important to note that maximum power values can be achieved through different combinations of F0 and V0^9^. Ideally, high values of both F0 and V0 are generally maintained throughout the sprint; however, at the static start of the sprint, a player produces a high amount of horizontal force at low velocity to overcome inertia. As the sprint progresses, velocity increases and horizontal forces decrease, while vertical forces increase as the player accelerates and covers greater distance^8^. Additionally, the F-V relationship is influenced by factors such as gender, weight, height, age, and level of practice^10^. In football training, it is essential to improve sprinting abilities. To achieve this, a thorough understanding of the factors influencing sprint performance, particularly F0, V0, Pmax, and F-V slope, is necessary to develop more accurate training programs and improve sprint performance.

In football, the F-V profile is commonly obtained by conducting a linear sprint on the field, during which the player reaches maximum velocity over a distance of at least 30 m; position, velocity, and time values are then analyzed^7,11^. Instruments such as lasers, radars, mobile apps with slow-motion video recording, and global navigation satellite systems (GNSS) can be used to measure this data^12^. Previous studies have evaluated the validity and reliability of GNSS devices and conducted research on football players^13^, concluding that these are valid and reliable instruments (Pearson evaluation values between 0.48 and 0.96 in the different profile variables) for obtaining the F-V profile in team sports players^14^.

Previous research has primarily analyzed the F-V profile in professional players throughout a season, where higher acceleration values were observed mid-season; however, values related to maximum velocity showed no changes^15^. Depending on the category of play and differentiating by gender, greater sprinting capacities were observed in men playing in higher categories, with smaller differences seen in women^9^.While the F-V profile is influenced by various factors across different populations, there is limited scientific evidence on how it evolves in children and adolescents depending on their age category or over the course of a season.

Therefore, the aim of this study was to analyze the changes in the F-V sprint profile and 30-meter sprint times of young players in a football academy throughout the three phases of the season within each age category, also differentiating between the different categories. The hypothesis was that the sprint strength-velocity profiles and 30-meter sprint times of youth soccer academy players will change significantly over the course of the season, with observable differences between age categories due to physical development and training adaptations.

## Materials and methods

### Subjects

A sample of 64 children and adolescents aged 10–18 years, from a football academy in Toledo, Spain, participated in this study. The sample power and criteria being adequate according to previous research^16^. All participants volunteered and were divided into five groups based on their playing category (U10, U12, U14, U16, U18). The general characteristics of each group are described in Table 1. Written informed consent was obtained from all children and their legal representatives. The study was approved by the Clinical Research Ethics Committee of the Castilla-La Mancha Health Service (Spain), following the latest version of the Declaration of Helsinki (Ref.: 489/24022020).

**Table 1:** Descriptive characteristics of football players by age category.

Measurement		U10	U12	U14	U16	U18
		(*n* = 13)	(*n* = 13)	(*n* = 17)	(*n* = 12)	(*n* = 9)
Age (years)	P1	9.6 ± 0.7	11.8 ± 0.8	13.6 ± 0.7	15.8 ± 0.7	17.6 ± 0.6
	P2	9.8 ± 0.8	11.3 ± 0.7	13.8 ± 0.8	15.6 ± 0.5	17.8 ± 0.4
	P3	10.6 ± 0.8	12.2 ± 0.6	14.5 ± 0.7	16.4 ± 0.7	18.5 ± 0.6
Height (cm)	P1	137.2 ± 18.1	143.7 ± 19.6	156.1 ± 36.7	170.3 ± 25.7	173.8 ± 20.4
	P2	138.7 ± 21.3	145.6 ± 19.4	159.2 ± 33.3	171.1 ± 25.2	173.1 ± 18.7
	P3	140.2 ± 25.1	147.3 ± 23.8	162.3 ± 32.8	172.4 ± 25.3	174.5 ± 19.6
Weight (kg)	P1	31.5 ± 12.7	37.3 ± 31.2	46.9 ± 27.5	61.7 ± 43.6	65.8 ± 27.6
	P2	32.7 ± 14.2	38.8 ± 31.8	48.1 ± 29.6	64.0 ± 44.7	64.3 ± 28.3
	P3	32.2 ± 12.3	39.7 ± 32.6	50.8 ± 31.7	64.2 ± 44.6	67.6 ± 23.1
Lean Body mass (g)	P1	23785.73 ± 2479.68	26529.42 ± 3345.42	36117.83 ± 5554.00	48662,24 ± 4674.31	52531,68 ± 5701.17
	P2	24274.78 ± 2473.20	26927.82 ± 3491.66	37418.91 ± 5994.39	48945.64 ± 4702.52	52554,58 ± 6893.87
	P3	25202.87 ± 2534.08	27997.01 ± 3664.72	39764.05 ± 6161.17	50145.35 ± 4780.38	52192,57 ± 54932.45
Body mass index (BMI: kg · m^–2^)	P1	16.4 ± 1.6	18.2 ± 2.3	18.8 ± 1.8	21.1 ± 2.6	21.8 ± 2.3
	P2	16.7 ± 1.7	18.3 ± 2.4	19.1 ± 1.9	21.9 ± 2.5	21.4 ± 2.2
	P3	16.2 ± 1.7	18.1 ± 2.5	19.1 ± 1.7	21.6 ± 2.7	22.2 ± 2.3
Competitive level		Provincial	Provincial	Regional	Regional	National
Previous experience (years)		2 ± 1.7	3 ± 2.3	4 ± 2.8	5 ± 3.1	11 ± 2.7
Training frequency (h/week)		2.5	2.5	3.5	3.5	5

Mean ± SD; cm = centimeters; kg = kilograms; m = meters; h = hours; g = grams.

### Experimental approach to the problem

A longitudinal study was designed to analyse the sprint F-V profile in male amateur football players at three points during the season: P1 (September 2021), P2 (January 2022), and P3 (May 2022). No specific interventions were implemented to increase or improve sprinting capacity at different testing points, other than regular training and match activities. All tests were conducted 48 h after a competition. Participants wore the same training clothes and shoes for each test, and the testing field remained consistent.

### Procedures

The F-V profile was assessed at the beginning of the afternoon training session at 18:30 (U10, U12, U14) and 20:30 (U16, U18) on an outdoor artificial turf football pitch. The warm-up lasted 20 min and included gentle continuous running, mobility exercises, and two progressive 30-meter sprints. After the warm-up, players performed two 30-meter sprints with a five-minute recovery period between each sprint. Players began in a crouched and staggered stance and were instructed to remain as still as possible to avoid any forward or backward movement before the sprint. A specific signal (“3, 2, 1, start the sprint”) was used to initiate the sprint, and players were instructed to accelerate maximally until reaching the 30-meter mark. Each sprint in each moment of the season was measured with a GNSS device (WIMU PRO™ RealTrack System SL, Almeria, Spain)^13^, which collected data at 10 Hz with a dual system (GNSS and GPS). Each player wore the device in a vest fitted to the torso. The time to complete the 30-meter sprint (s) was calculated using four pairs of Microgate Witty (Wireless Training Timer) photocells (software 2.17.4, Microgate Corporation, Bolzano, Italy), placed at 10, 20 and 30 m from the starting point. Participants started from a standing position on the starting line and sprinted the 30-meter distance as fast as possible until crossing the finish line. The time taken to cover the 30 m, as well as partial times at 10 and 20 m, were recorded.

The raw velocity data collected by the GNSS devices were downloaded using SPro software (Realtrack Systems SL, Almeria, Spain). Data were loaded into a custom-made script to automatically calculate F-V profiles (F0 (N), F0 (N·kg^–1^), V0 (m·s^–1^), Pmax (W), Pmax (W·kg^–1^), F-V slope, RF peak, and DRF) and times at 5, 10, 15, 20, and 30 m, based on the validated calculation methods from previous studies using raw velocity data (km·h^–1^) and time (seconds)^6^.

### Anthropometric and body composition measurements

Weight and height measurements were taken at the beginning of P1, P2, and P3. Body mass (kg) was measured using an electronic scale (Seca 813, Hamburg, Germany), and height (cm) was measured with a scientific height rod (Seca 214, Hamburg, Germany). Body mass index (BMI) was calculated for each participant using these measurements and expressed in kg·m^–2^. Lean Body mass (g) were estimated using dual X-ray absorptiometry (Hologic Series Discovery QDR, Software Physician’s Viewer APEX System Software Version 3.1.2, Bedford, MA, USA).

### Statistical analysis

The results are presented as mean ± standard deviations. Shapiro-Wilk and Levene’s tests were used to confirm normality of the data and homogeneity of variance (*p* > 0.05). Sprint F-V profile and sprint performance variables were compared using a two-way mixed ANOVA (with age category as a between-subject factor and time-points as a within-subject factor). The Bonferroni post-hoc test was applied for pairwise comparisons. A 95% confidence interval was included, and effect size (ES) was calculated using Cohen’s d and defined as follows: trivial (ES < 0.19), small (ES = 0.20–0.49), medium (ES = 0.50–0.79), and large (ES > 0.8). The level of significance was set at *p* < 0.05. All analyses were performed using the SPSS statistical package (v24, SPSS Inc., Chicago, IL, USA).

## Results

### Changes in force-velocity profile over time

The results for the different magnitudes of the force-velocity profile are presented in Table 2. F0 (N) was significantly higher in the third period compared to the first for U14 (*p* = 0.042; ES: 1.92), U16 (*p* = 0.001; ES: 2.81), and U18 (*p* < 0.001; ES: 5.19). F0 (N·kg-1) increased in U16 and U18 players (*p* < 0.001; ES: 5.19; *p* < 0.001; ES: 4.77). V0 (m·s-1) was higher in the third period for U12 (*p* = 0.012; ES: 2.61) and U16 (*p* = 0.024; ES: 2.37), but lower in U16 and U18 players between the first and second periods (*p* = 0.010; ES: 4.06; *p* = 0.013; ES: 2.80). FV-slope·kg-1 increased in U16 players (*p* = 0.048; ES: 0.28), but decreased in U18 players (*p* < 0.001; ES: 0.45).

**Table 2:** Comparison of the sprint force-velocity profile between the different time points of the season and age categories.

	Period 1					Period 2					Period 3				
	U10	U12	U14	U16	U18	U10	U12	U14	U16	U18	U10	U12	U14	U16	U18
F_0_ (N)	195.6 ± 19.6bcd	241.7 ± 19.5bcd	323.0 ± 17.1#	344.6 ± 20.4*#	356.6 ± 23.6*#	192.6 ± 20.2bcd	223.289 ± 22.258bcd	307.2 ± 17.7#cd	442.5 ± 21.1#	463.7 ± 24.3	208.8 ± 18.1bcd	247.1 ± 19.2bcd	354.9 ± 15.9d	400.1 ± 18.9	474.6 ± 21.8
F_0_ (N/kg^−1^)	6.1 ± 0.2	6.4 ± 0.2c	6.8 ± 0.2 cd	5.3 ± 0.2*#	5.4 ± 0.2*#	5.8 ± 0.2	5.7 ± 0.2	6.3 ± 0.1#	6.6 ± 0.2	6.7 ± 0.2	6.3 ± 0.2	6.3 ± 0.2	7.0 ± 0.2	6.1 ± 0.2	6.8 ± 0.3
V_O_ (m/s^−1^)	6.1 ± 0.1bcd	6.4 ± 0.1#bcd	7.3 ± 0.1 cd	9.0 ± 0.1*	9.2 ± 0.2*	6.3 ± 0.1bcd	6.3 ± 0.1#bcd	7.3 ± 0.1 cd	8.3 ± 0.1#	8.6 ± 0.2	6.3 ± 0.1bcd	6.8 ± 0.1bcd	7.6 ± 0.1 cd	8.7 ± 0.1	8.8 ± 01
P _max_ (W)	300.2 ± 36.4bcd	389.5 ± 38.9bcd	594.8 ± 31.9#cd	762.3 ± 37.9*#	817.6 ± 43.8*#	302.3 ± 43.4bcd	353.8 ± 41.2#bcd	568.1 ± 38.0#cd	920.1 ± 45.2	1003.3 ± 52.2	334.4 ± 39.2bcd	424.524 ± 37.284bcd	678.234 ± 34.337 cd	868.823 ± 40.870	1051.450 ± 47.192
P _max_ (W·kg^−1^)	9.5 ± 0.4bcd	10.3 ± 0.4*b	12.6 ± 0.4	11.9 ± 0.4*#	12.4 ± 0.5*#	9.1 ± 0.4bcd	9.2 ± 0.4#bcd	11.6 ± 0.4#cd	13.7 ± 0.5	14.5 ± 0.5	10.1 ± 0.5bcd	10.890 ± 0.503bcd	13.459 ± 0.489	13.277 ± 0.582	15.249 ± 0.672
FV-slope kg^−1^	− 1.01 ± 0.03 cd	-0.99 ± 0.03 cd	-0.93 ± 0.03 cd	-0.61 ± 0.03*	− 0.59 ± 0.04*#	− 0.92 ± 0.03	− 0.91 ± 0.02	-0.86 ± 0.02	− 0.80 ± 0.03#	− 0.78 ± 0.04	-0.99 ± 0.03 cd	− 0.92 ± 0.04c	− 0.92 ± 0.03c	− 0.70 ± 0.04	− 0.77 ± 0.04
RF_peak_ (%)	16.4 ± 0.4bcd	16.7 ± 0.5#bcd	19.1 ± 0.4 cd	23.1 ± 0.4	24.0 ± 0.5	16.6 ± 0.5bcd	16.9 ± 0.5#bcd	1.95 ± 0.4 cd	22.1 ± 0.5#	23.0 ± 0.6	16.4 ± 0.5bcd	18.2 ± 0.4 cd	19.8 ± 0.4 cd	23.3 ± 0.5	23.8 ± 0.5
D_RF_ (%)	10.0 ± 0.4 cd	9.8 ± 0.3 cd	9.2 ± 0.3 cd	5.9 ± 0.4*	5.7 ± 0.4*#	9.2 ± 0.3d	9.0 ± 0.4	8.5 ± 0.3	7.8 ± 0.4#	7.6 ± 0.4	9.9 ± 0.3 cd	9.1 ± 0.3 cd	9.1 ± 0.3 cd	6.7 ± 0.004c	7.5 ± 0.4

Notes: Differences concerning the mentioned group at a (U12). b (U14). c (U16). d (U18) *P* < 0.05. Differences with respect to the mentioned time in * (moment 2). # (moment 3) *P* < 0.05. g: grams; %: percentage. N: newton; F: force; kg: kilograms; P: peak; W: watios; FV: force-velocity; %: percentage; F0: theoretical maximal force; V0: theoretical maximal velocity; FV slope: force-velocity slope; Pmax: theoretical maximal power; DRF: decrease in the ratio of horizontal force; RF peak: maximal ratio of horizontal force.

### Differences in force-velocity profile between categories

U10 and U12 players had lower F0 (N) values than U14, U16, and U18 players (*p* < 0.05; ES: 4.41–13.38). F0 (N·kg-1) was higher in U12 compared to U16 (*p* = 0.040; ES: 4.23), and U14 had higher values than U16 (*p* < 0.001; ES: 6.45) and U18 (*p* = 0.002; ES: 5.75). U10 and U12 players consistently had lower V0 (m·s-1) and Pmax (W) than older categories (*p* < 0.05; ES: 1.68–5.64). FV-slope·kg-1 was higher in U16 and U18 compared to younger categories (*p* < 0.01; ES: 9.84–11.02).

### Difference in sprint time across moments

**Fig. 1.**
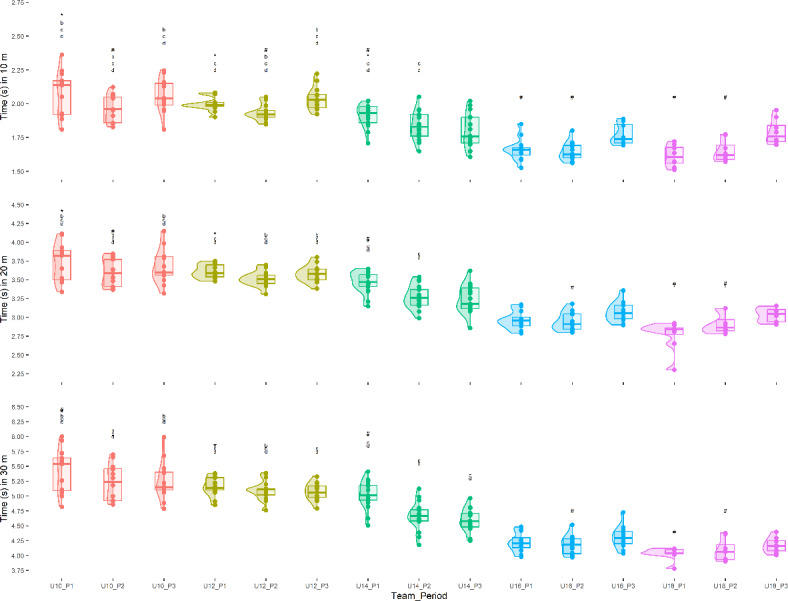
Sprint time difference between categories and across moments at 10, 20 and 30 m.

The results for the 30-meter sprint times are presented in Fig. 1. U10, U12 and U14 players required more time to cover the first 10 m in the first period compared to the second (*p* < 0.05; ES: 0.88–1.09). U16 and U18 players showed shorter execution times in the third period compared to the first for the first 10 m (*p* < 0.019; ES: 0.25; *p* < 0.001; ES: 2.00). In the 20-meter leg, U10, U12 and U14 players had longer times in the first period (*p* < 0.05; ES: 0.60–1.37), while U14 and U18 players achieved shorter times in the first period relative to the third (*p* < 0.001; ES: 1.43–1.55). U12 and U14 players completed the 30 m in less time in the third period compared to the first (*p* < 0.05; ES: 0.64–2.09).

### Difference in sprint time between categories

U10 and U12 players required more time to cover 10, 20 and 30 m than U14, U16 and U18 players in all three periods (*p* < 0.05; ES: 1.05–2.48). U14 players required more time to cover the first 10 m than U16 and U18 players in the first two periods (*p* < 0.05; ES: 0.38–1.28). U12 and U14 players had longer times to cover the 30 m compared to U16 and U18 players in all three periods (*p* < 0.05; ES: 0.74–2.36).

## Discussion

The aim of this research was to analyse the changes over the course of a season (start, mid-season, and end) within each age category, also differentiating between the different categories in the F-V sprint profile and 30-metre sprint times of young players in a football academy. This is the first study to conduct a all-season longitudinal observation of this profile across different categories and points in the season in a youth population. The longitudinal observation yielded significant results between the different categories. This research revealed significant differences between U10 and U12 players and U14, U16, and U18 players in variables such as horizontal force (F0), maximum velocity (V0), and the F-V slope. Additionally, fluctuations were observed throughout the season, with a decrease in force and velocity values mid-season, followed by an increase at the end of the season. These changes were consistent across all age categories in both the F-V profile and 30-metre sprint times.

In terms of F0 magnitude, players demonstrated a progressive increase in horizontal force exerted during sprinting, depending on the category. U10 and U12 players had significantly lower results than U14, U16, and U18 players. These differences may be attributed to muscle mass^17–19^; greater muscle mass allows for more force generation and higher F0 values. Previous longitudinal studies in adults have shown different results, with F0 values peaking mid-season and declining at the end^15^. However, the evolution of F0 throughout the season in children shows fluctuations, with higher values at the beginning and end of the season than in the middle. The timing of the evaluations is crucial, as even in a short period like a match, they can vary^20^. The decrease in F0 mid-season could be attributed to evaluations conducted weeks after a holiday period, which caused a decrease in strength, which later increased by the end of the season with resumed football training.

Regarding V0, U16 and U18 players had significantly higher V0 values than U10, U12, and U14 players throughout the season. This may be due to increased strength and mechanical factor development between ages 16 and 18; greater stride length and frequency, better coordination and body stabilisation, and shorter ground contact times contribute to increased velocity^21–23^. The evolution of V0 throughout the season mirrors F0, with a significant decrease in V0 values mid-season, especially in U16 and U18, followed by an increase at the season’s end. The cause could be similar to F0, where the holiday period following the second measurement could have caused a decrease in values. However, at the season’s end, V0 increases in U10, U12, and U14 players but decreases in U16 and U18 players, with these categories showing their highest V0 at the season’s start. The absence of significant improvements in U16 and U18 may be due to stabilised strength and developed mechanical factors, where football training does not enhance this magnitude when performing sprints that do not achieve or maintain maximum velocity^24,25^. However, previous studies in professional football players have shown a progressive increase in V0 values throughout the season^15^.

The relationship between strength and velocity is observed in the FV-slope-kg-1, which shows significant differences between U10 and U12 players, where strength predominates in sprint performance, while speed predominates in U16 and U18 players. This may be due to the physical demands of training and matches. U10 and U12 players play 8-a-side soccer with fewer square meters per player and a greater number of actions requiring higher levels of strength, such as accelerations and decelerations^26^. However, U16 and U18 players play on a larger field with more square metres per player, exhibiting more high-velocity actions^27^. On the other hand, previous studies showed that the maturational stage significantly influences the force-velocity (F-V) profile in football players, particularly in young athletes undergoing different stages of growth. Research shows that players at different maturational stages—classified as pre-, mid-, and post-peak height velocity (PHV)—exhibit distinct characteristics in their sprinting performance and F-V profiles. Pre-PHV players show greater improvements in sprint acceleration-related components, such as maximal theoretical force (F0) and the ratio of horizontal-to-resultant force (RFpeak), compared to their mid-PHV counterparts. Conversely, post-PHV players demonstrate significant enhancements in peak velocity-related components, such as maximal theoretical velocity (V0) and the decrease in the ratio of horizontal-to-resultant force (DRF)^22^. This indicates that strength development is more pronounced in earlier maturation stages, while velocity development accelerates in later stages. Training programmes for young soccer players should be tailored to these developmental differences to optimise performance outcomes.

Regarding the study’s limitations and potential future research directions, it is important to note that the research was conducted exclusively on amateur teams from a single academy, which limits the ability to generalise the findings to other levels of competition or contexts. Future studies should include more diverse samples, considering academies from different regions, elite levels and international competitions. By not including specific interventions, it is not possible to establish causal relationships between measured variables and changes in the F-V profile. Future research could implement training protocols specifically designed to improve strength, velocity and their interaction in different categories. The analysis was based on age categories and not on stages of biological maturation or growth, which could limit the understanding of the impact of developmental phases on physical abilities. Including maturation indicators such as peak age of growth velocity (PHV) would provide a more accurate approach. Elements such as sleep quality, diet and academic stress, which also affect performance, were not considered in this study, which could have influenced the observed results. Climatic aspects such as wind were also not considered, which could influence the different shots when performing the sprint. In future research, the integration of AI methodologies could open new opportunities to optimize the analysis and application of these findings through predictive analytics, advanced personalisation and real-time profile analysis.

## Conclusions

The F-V profile throughout a season in young players of a football academy shows that theoretical maximum strength and velocity values increase in all categories after nine months of football training. However, holiday periods or breaks in football practice negatively affect the profile. Concerning category-based results, both theoretical maximum strength and velocity, and their derived variables, are higher in U16 and U18 players. These findings can be useful for coaches in training academies, particularly in the youth academies of professional clubs, to consider possible changes in the strength-velocity profile of their players and develop training programs focusing on improving these physical qualities according to the player’s category, preparing them for future demands and thus preventing future injuries due to strength or velocity deficits.

## Data Availability

The data supporting the findings of this study are available from the corresponding author upon reasonable request. Due to ethical and privacy concerns, the data cannot be made publicly available. Anonymized datasets can be shared with qualified researchers under a data use agreement approved by the institutional ethics committee.
